# Death after Sexual Intercourse

**DOI:** 10.1155/2015/646438

**Published:** 2015-12-01

**Authors:** Christian T. Braun, Meret E. Ricklin, Andreina Pauli, Daniel Ott, Aristomenis K. Exadaktylos, Carmen A. Pfortmueller

**Affiliations:** ^1^Department of Emergency Medicine, Inselspital, University Hospital Bern, Freiburgstrasse 10, 3010 Bern, Switzerland; ^2^University Institute of Diagnostic, Interventional and Pediatric Radiology, Inselspital, University Hospital Bern, Freiburgstrasse 10, 3010 Bern, Switzerland; ^3^Department of General Anesthesiology, Intensive Care and Pain Management, Medical University of Vienna, Waehringerguertel 18-22, 1090 Vienna, Austria

## Abstract

Sexuality is an essential aspect of quality of life. Nevertheless, sexual intercourse is physically challenging and leads to distinct changes in blood pressure, heart, and respiratory rate that may lead to vital complications. We present a case report of a 22-year-old female suffering from subarachnoid hemorrhage after sexual intercourse. The patient was immediately transported to hospital by emergency medical services and, after diagnosis, transferred to a tertiary hospital with neurosurgical expertise but died within 24 hours. After postcoital headaches, subarachnoid hemorrhage is the second most common cause of neurological complications of sexual intercourse and therefore patients admitted to an emergency department with headache after sexual intercourse should always be carefully evaluated by cerebral imaging.

## 1. Introduction

Sexuality is an essential aspect of quality of life [1–3]. Nevertheless, sexual intercourse is physically challenging and leads to distinct changes in blood pressure, heart, and respiratory rate [3, 4]; for an overview see Table 1.

The complications of sexual intercourse are apparently rare relative to the frequency of coitus in the general population [3, 5, 6]. The true incidence is not known, as patients may not report the sexual circumstances of their health problems to health care professionals [5, 6]. Nevertheless, it has been reported that sexual intercourse may lead to severe injury [3, 4].

## 2. Case Presentation

We present a case of a 22-year-old female admitted unconscious to the emergency department by emergency medical services. The medical history revealed that the woman suffered from a seizure followed by unconsciousness while having sexual intercourse. The patient had never had a seizure before and had no prior history of headaches, migraine, head trauma, substance abuse, or intoxication. The patient had previously been in excellent health and with physical fitness appropriate to her age. Her only regular medication was an oral anticontraceptive.

**Figure 1:**
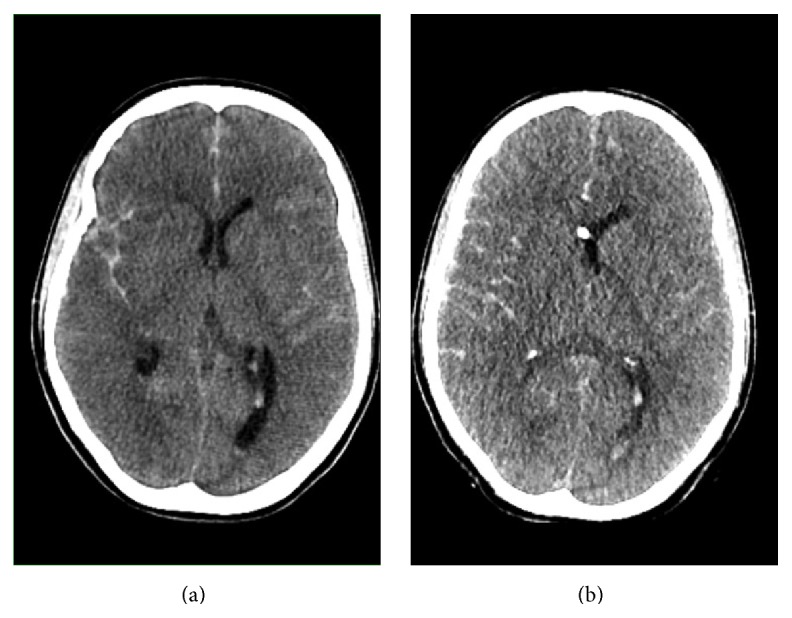
(a) First computed tomography of the head (01:25 am) and (b) follow-up computed tomography of the head (02:30 am).

On admission to the emergency department (ED) of a secondary care hospital, the clinical findings were as follows: unconsciousness with a Glasgow Coma Scale (GCS) of 3 and unilaterally (left) light reactive pupil; no seizure was observed. The patient was breathing spontaneously through a nasal cannula, with a normal respiratory rate and 4l oxygen saturation. Blood pressure, heart rate, and temperature were normal. After immediate airway management, computed tomography (CT) of the head was performed. She was found to have an extensive subarachnoid hemorrhage (Fisher grade 4), with breach in the fourth ventricle; see Figure 1. Clinical examination and laboratory analysis did not reveal any further pathologic features. The patient was urgently transferred by helicopter to our tertiary hospital for neurosurgical intervention.

**Figure 2:**
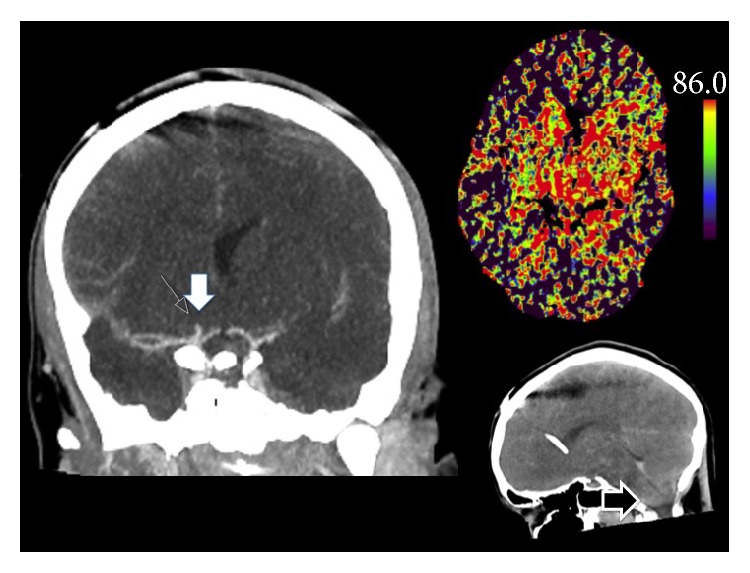
Second computed tomography of the head (02:30 am) shows an aneurysm at the right carotid T cross, pan-cerebral diminished perfusion, and developing tonsillar herniation.

On admission to our emergency department (ED), clinical examination showed bilaterally wide pupils with bilaterally absent pupillary reflexes, irregularities in the pupillary margin, and absent brain stem reflexes; for a timeline of changes in neurological findings see Table 2. A ventricle drainage was immediately placed and showed an opening pressure of 86 mmHg. Thus, conservative measurements to lower intracerebral pressure were started (head up placement, hyperventilation, intravenous mannitol, maximal sedation, and muscle relaxation) and computed tomography with angiography was performed. This showed an extensive and progressive subarachnoid hemorrhage (now Fisher grade 4) with diminished perfusion during the arterial phase, collapsed ventricles, cerebral herniation, pan-cerebrally diminished perfusion, and a potential aneurysm at the carotid artery cross; see Figure 2. Despite maximal conservative therapy (see above) a decline in cerebral pressure could not be achieved.

After careful consideration by all specialists involved and with the consent of the patient's relatives, it was decided that further neurosurgery would not be performed, as the brain stem reflexes were extinct and the intracerebral pressure had remained at 80 mmHg for more than one hour despite maximal conservative therapy. The patient was transferred to the intensive care unit for organ saving therapy and died 48 hours after admission.

## 3. Discussion

Our case features sexual intercourse as a trigger of an acute intracerebral hemorrhage in a young female. Several studies have shown that sexual intercourse may provoke intracerebral hemorrhage, especially subarachnoid hemorrhage [3, 4]. It has been reported that 14.5% of all subarachnoid hemorrhages are precipitated by sexual activity [7, 8]. Our patients most likely had a preexisting vascular aneurysm as a precipitating lesion for subarachnoid bleeding, as the second computed tomography showed. It has been reported that the acute elevation in blood pressure during sexual intercourse increases the vessels' wall tension and the subsequent risk of its rupture by 15-fold [4, 9]. Nevertheless, this connection has only been seen in the few existing observational studies on this topic; further scientific evaluation of the cohesion between sexual intercourse and cerebral aneurysm rupture should be performed.

Although the published literature is sparse on the topic of sexual intercourse-related subarachnoid hemorrhage, several studies have found a male predominance [7, 9–11]. This is striking for two reasons: firstly it is known that the incidence of cerebral aneurysms is higher in females [12] and secondly as women may experience multiple and longer orgasms than men, it would be expected that wall tension in cerebral vessels would be elevated for longer than in males [9].

Patients with sexually triggered subarachnoid hemorrhage most often present with severe headache [3, 4]. Headaches are the most common symptoms and pathology of patients presenting with sexual intercourse-related problems to the emergency department [3], amounting to almost 50% of the total [14]. The explosive character of coital headache makes it difficult to differentiate from more severe disease [6]; therefore, subarachnoid hemorrhage and arterial dissection should always be excluded by radiological image study [6, 15, 16]. The pathophysiology of orgasmic headache is not yet completely understood [15, 16]; arterial vasospasm secondary to impaired myogenic cerebral autoregulation may play a role [8, 17, 18].

In contrast to the overall frequency of headaches in sexual intercourse-related admissions to the emergency department, our patient suffered a seizure followed by unconsciousness. Her seizure was certainly due to the massive subarachnoid hemorrhage. Nonetheless, in rare cases, epileptic seizures are induced by sexual orgasm [19]. They are predominant in females and their origin is thought to be in the right hemisphere [19, 20].

Unfortunately the patient featured in our case report died. Death due to sexual intercourse is rare [3, 4]. In two large autopsy studies with 5559 patients, the rate of death due to sexual intercourse was estimated to be about 0.6% [10, 21]. Male gender and extramarital sexual activity as well as excessive alcohol consumption and large meals are a risk factor for death related to intercourse [1, 22, 23].

## 4. Conclusion

In conclusion, sexual intercourse might be a precipitating factor for subarachnoid hemorrhage with a potentially fatal outcome, as this case report shows. Nonetheless, further studies are needed to prove a direct relationship between the sexual intercourse and cerebral aneurysm rupture. After excluding intracerebral pathologies such as subarachnoid hemorrhage or intracranial bleeding by cerebral computed tomography, further differential diagnosis should involve seizures triggered by postcoital headaches and sexual intercourse.

## Figures and Tables

**Table 1:** Overview on stages of the sexual cycle [3, 13].

Stage	Explanation	Body's response
1	Excitement (initial state of arousal)	Increases in muscular tone, heart rate, and blood pressure
2	Plateau (full arousal immediately preceding orgasm)	Further increases in muscular tone, heart rate, and blood pressure and increased relative vascular resistance
3	Orgasm	Associated with muscle spasms, massive elevation of heart rate, blood pressure, and respiratory rate
4	Resolution	Normalization of physical function

**Table 2:** Timeline of neurological findings.

	00:35	00:55	01:25	02:30	08:00
Event	Sexual intercourse	Ambulance arrived	Arrival at secondary care hospital	Arrival at tertiary care hospital	Neurological re-evaluation on ICU

Pupils	Normal	Miotic bilaterally light reactive pupils	Unilaterally light reactive pupil (left), right pupil wide and nonreactive	Bilaterally wide pupils with absent pupillary reflexes bilaterally, irregularities to pupillary margin	Bilaterally wide pupils with absent pupillary reflexes bilaterally, irregularities to pupillary margin

Brainstem reflexes	Present	Present	Unclear	Lack of brainstem reflexes	Lack of brainstem reflexes

CT	—	—	Figure 1	Figures 1 and 2	—

Intracerebral pressure	—	—	—	86 mmHg	90 mmHg

Other findings	Generalized seizure, comatose			Lack of corneal reflexes	Lack of corneal reflexes
